# Composited Film of Poly(3,4-ethylenedioxythiophene) and Graphene Oxide as Hole Transport Layer in Perovskite Solar Cells

**DOI:** 10.3390/polym13223895

**Published:** 2021-11-11

**Authors:** Tian Yuan, Jin Li, Shimin Wang

**Affiliations:** Hubei Collaborative Innovation Center for Advanced Organic Chemical Materials, Ministry of Education Key Laboratory for the Green Preparation and Application of Functional Materials, School of Materials Science and Engineering, Hubei University, Wuhan 430062, China; tianyuan@stu.hubu.edu.cn

**Keywords:** perovskite solar cell, hole transport layer, carbon materials, polymeric composites, solar energy materials

## Abstract

It is important to lower the cost and stability of the organic–inorganic hybrid perovskite solar cells (PSCs) for industrial application. The commonly used hole transport materials (HTMs) such as Spiro-OMeTAD, poly[bis(4-phenyl)(2,4,6-trimethylphenyl)amine] (PTAA) and poly(3-hexylthiophene-2,5-diyl) (P3HT) are very expensive. Here, 3,4-ethylenedioxythiophene (EDOT) monomers are in-situ polymerized on the surface of graphene oxide (GO) as PEDOT-GO film. Compared to frequently used polystyrene sulfonic acid (PSS), GO avoids the corrosion of the perovskite and the use of H_2_O solvent. The composite PEDOT-GO film is between carbon pair electrode and perovskite layer as hole transport layer (HTL). The highest power conversion efficiency (PCE) is 14.09%.

## 1. Introduction

Renewable clean energy devices are urgently demanded for the sustainable development of society. Among them, organic–inorganic metal hybrid perovskite solar cells (PSCs) have attracted ever-increasing attention owing to their excellent photovoltaic performance, simple preparation process, and relatively low cost [1]. PSCs are generally composed of FTO glass, an electron transport layer, a light absorption layer, a hole transport layer, and a counter electrode [2]. In the multi-layer structure of PSCs, the hole transport layer (HTL) is designed to promote the separation of electrons and holes, which is key to the performance and stability of the cell. However, certain problems of HTL hinder the development and application of the PSCs technology. Currently, the HTL of PSCs are based on materials such as Spiro-OMeTAD, PTAA [3] and P3HT [4]. The costs of these materials are all prohibitively high for large-scale applications [5]. What is more, the dopants in Spiro-OMeTAD show strong water absorbency, which seriously threatens the service life of PSCs [6]. Therefore, it is necessary to explore a low-cost and stable hole transport materials (HTMs) for the practical stage of PSCs.

PEDOT, usually combined with PSS, is widely used in inverted PSCs [7,8,9], whose price is much cheaper than the materials mentioned above. However, sulfonic acid groups contained in PSS are extremely harmful to the device life. To avoid the usage of PSS, Jiang et al. [10] synthesized 2,5-dibromo-3,4-ethylenedioxythiophene (DBEDOT) monomer, which was spin-coated on a perovskite layer and in-situ polymerized as PEDOT; a photoelectric conversion efficiency (PCE) of PSCs of about 17% was achieved. Wei et al. [11] used sulfonated acetone-formaldehyde (SAF), instead of PSS, to composite with PEDOT in inverted PSCs, which effectively increased the life of PSCs. Meanwhile, graphene oxide (GO) was also selected as the HTM of PSCs. Wu et al. [12] fabricated 2 nm thickness GO film as HTL, and the PCE of the inverted PSCs reached 12.40%. 

In this work, a harmless HTL was obtained by PEDOT composited with GO. The PEDOT interacted with GO sheet via π–π stacking and hydrogen-bonding interactions, thus a conjugated system can be formed [13]. Moreover, GO functions as an excellent carrier to enable the dispersion of PEDOT in isopropanol solution, which is also harmless to the perovskite layer. Using PEDOT-GO film as HTL, a PSC with a PCE of up to 14.09% with good stability can be realized based on carbon counter electrode.

## 2. Materials and Methods

### 2.1. Material Preparation

The perovskite (PbI_2_, MAI), FTO glasses (3 × 3 cm^2^) and the hole transport layer (HTL) ((Spiro-MeOTAD, lithium-bis (tri-fluoromethanesulfonyl) imide (Li-TFSI), and 4-tert-butylpyridine (tBP)) solution were purchased from Xi'an Polymer Light Tech-nology Co., Ltd. (Xian,China) 3,4-ethylenedioxythiophene (EDOT), graphite, acidic (NH_4_)_2_S_2_O_8_ (APS), TiCl_4_, DMSO (99.9%) and 4-hydroxybutyric acid lactone (DMF) (99.9%) were purchased from Aladdin. Acetone, ethanol, and isopropyl alcohol were purchased from Sinopharm Chemical Reagent Co., Ltd. (Sinopharm Chemical Reagent Co., Ltd. ) And all the materials were used as received without further purification.

### 2.2. Fabrication of Device

FTO glasses were ultrasonically cleaned with detergent, acetone, ethanol, and isopropyl alcohol sequentially. After that, they were dried under hot air and treated in ultraviolet-ozone for 15 min. The clean FTO substrate was then soaked in dilute 0.2 M aqueous TiCl_4_ solution at 70 °C for 1 h, and washed with deionized water, then annealed at 200 °C for 60 min. In the Glove box, dropping the perovskite (CH_3_NH_3_I: 159 mg, PbI_2_: 461 mg, DMF: 600 mg, and DMSO: 78 mg) onto the substrate and deposited it by spin-coating at 4000 rpm for 30 s. Solvent treatment was conducted at late 15 s, where 150 μL chlorobenzene was dropped on the spinning substrate followed by annealing at 100 °C for 10 min. After cooling to room temperature, the hole transport materials prepared before (3 mg/mL in dimethylcarbinol) were spin-coated onto the perovskite layer at 2000 rpm for 30 s and followed by 10 min of thermal annealing at 90 °C. Besides, Spiro-OMeTAD hole transport material was used in the comparative experiment: 20 μL spiro-OMeTAD solution, containing 36.1 mg spiro-OMeTAD, 14.4 μL t-BP and 9 μL Li-TFSI solution (520 mg in acetonitrile), was spin-coated on the perovskite layer at 4000 rpm for 30 s. Lastly, a carbon black counter electrode was coated on the top of the device by blade coating and sintered at 80 °C for 30 min.

### 2.3. Device Characterization

The X-ray diffraction (XRD) patterns of the samples and perovskite films were measured with a Bruker-AXS D8 Advance (Malvern Panalytical, Malvern, UK). MAPbI_3_ perovskite films morphology was measured with scanning electron microscope (SEM, sigma 500, Krefeld, Germany). The GO nanosheets were characterized via transmission electron microscopy (TEM, TecnaiG2 F20, FEI Company, Hillsboro, OR, USA). The photocurrent-voltage (J-V) characteristics of PSCs were analyzed under simulated AM 1.5 G radiation (100 mW/cm^2^ irradiance) by using a solar simulator (Oriel, model 91192-1000) and a source meter (Keithley 2400, USA). Electrochemical impedance spectroscopy (EIS) was measured with an electrochemical workstation (Zennium, IM6, Kronach, Germany) over the frequency range of 100 mHz^−2^ MHz with 10 mV AC amplitude at −1 V bias under simulated AM 1.5 G radiation (100 mW/cm^2^ irradiance). The steady-state photoluminescence (PL) measurements were acquired using an Edinburgh Instruments FLS920 fluorescence spectrometer (Oxford Instruments, Abingdon, UK). Raman spectroscopy analysis was performed by a micro-Raman instrument (XperRam 200, Nanobase, Seoul, South Korea), using 542 nm excitation with an incident power of 5 mW. The devices were measured under ambient conditions (15% < relative humidity (RH) < 60%) every time (winter, summer). After the measurements, the devices were stored in a humidity-controlled dry room (20% < RH < 40%).

## 3. Results and Discussion

**Figure 1:**
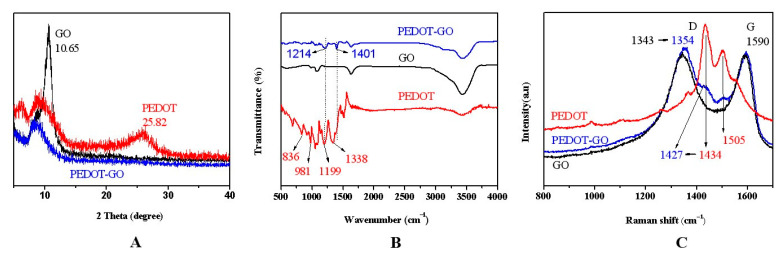
(**A**) XRD of GO, PEDOT and PEDOT-GO (**B**) FTIR spectra of GO, PEDOT and PEDOT-GO (**C**) Raman spectra of GO, PEDOT and PEDOT-GO.

Figure 1A–C respectively correspond to XRD, FT-IR, and Raman analyses to explore the structural properties of the as-prepared materials [14,15,16]. As shown in Figure 1A, an intense and sharp peak centered at 10.65° in the (GO) curve, which corresponds to the (001) crystal surface of the GO nanoflakes. The pattern of pure PEDOT depicts a broad peak in the region of 25.82°, which corresponds to the polymer chain structure of PEDOT. However, neither of the two peaks appeared in the composite samples. This is due to the influence of conjugation and the coating effect of PEDOT on GO sheets, indicating that the in-situ polymerization changes the growth state of the polymer chain [14,17].

In Figure 1B, the PEDOT curve shows two peaks at 981 cm^−1^ and 836 cm^−1^ are duo to C–S–C bond stretching of thiophene ring. The tensile vibration of the C–O–C bond at 1199 cm^−1^ was detected. The peak at 1338 cm^−1^ is due to C=C and C–C in the thiophene ring indicating that PEDOT was successfully synthesized. Combining three curves, the characteristic peaks of GO and PEDOT are all reflected in PEDOT-GO. Furthermore, two peaks at 1199 cm^−1^ and 1338 cm^−1^ on PEDOT skewed to 1214 cm^−1^ and 1401 cm^−1^ on the curve PEDOT-GO. This redshift phenomenon was due to the π–π stacking interaction between GO and PEDOT [13].

In Raman spectra, the three characteristic peaks of 441 cm^−1^, 1434 cm^−1^, and 1505 cm^−1^ in the red curve indicate the successful synthesis of PEDOT [18]. Meanwhile, the characteristic peaks of 1343 cm^−1^ and 1590 cm^−1^ (black curve) correspond to the respiratory vibration peaks of SP2 hybrid carbon atoms and the symmetric stretching motion peaks of SP2 hybrid atoms in the carbon ring, respectively, which are the characteristic peaks (D and G) of GO. Meanwhile, the characteristic peaks at 1434 cm^−1^ (red curve) are assigned to C_α_ = C_β_ symmetric stretching vibration in PEDOT, which moves to 1427 cm^−1^ in the Raman spectra of the PEDOT-GO sample (Figure 1C). This redshift phenomenon demonstrates that the PEDOT polymer changed to the quinoid form, and thus enabled the increase of conductivity [19]. Moreover, the characteristic peaks of GO and PEDOT are reflected in the curve of PEDOT-GO, confirming the in-situ polymerization of PEDOT-GO nanocomposites.

**Figure 2:**
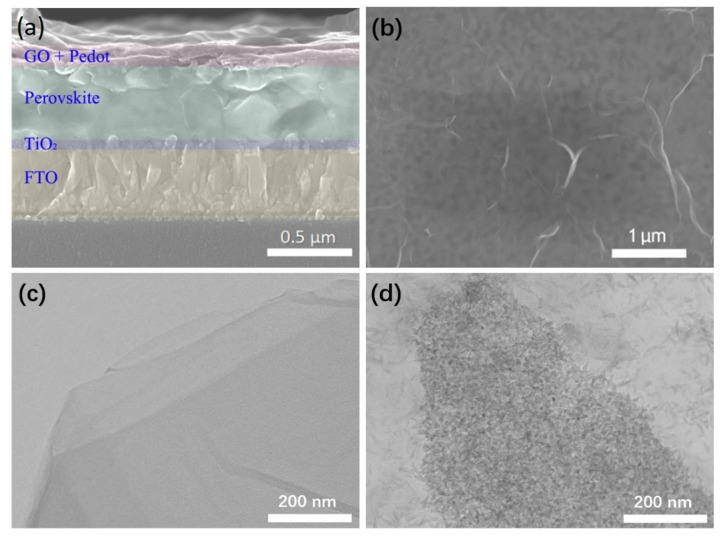
(**a**,**b**) SEM images of the device (**c**) TEM images of GO (**d**) TEM images of PEDOT-GO.

Figure 2a depicts an SEM cross-sectional view of the device. The cell structure is clearly displayed, and the thickness of the composite film is about 50 nm. Figure 2b presents the top view of the device [20,21,22]. The entire composite film is thin and evenly covered on the perovskite layer. Figure 2c,d are the TEM images of the GO and PEDOT-GO composite material, respectively. It can be seen that the surface of the graphene oxide sheet is smooth and transparent (Figure 2c). By comparison, the surface of the graphene oxide sheet is coated with a large amount of PEDOT nanoparticles in the PEDOT-GO film (Figure 2d), which is mutually confirmed with the previous analysis. This tight combination comes from the presence of conjugated heterocyclic structures and electronegative oxygen atoms. Simultaneously, PEDOT rich in free electrons and GO rich in carboxyl group form a good conjugated structure.

**Figure 3:**
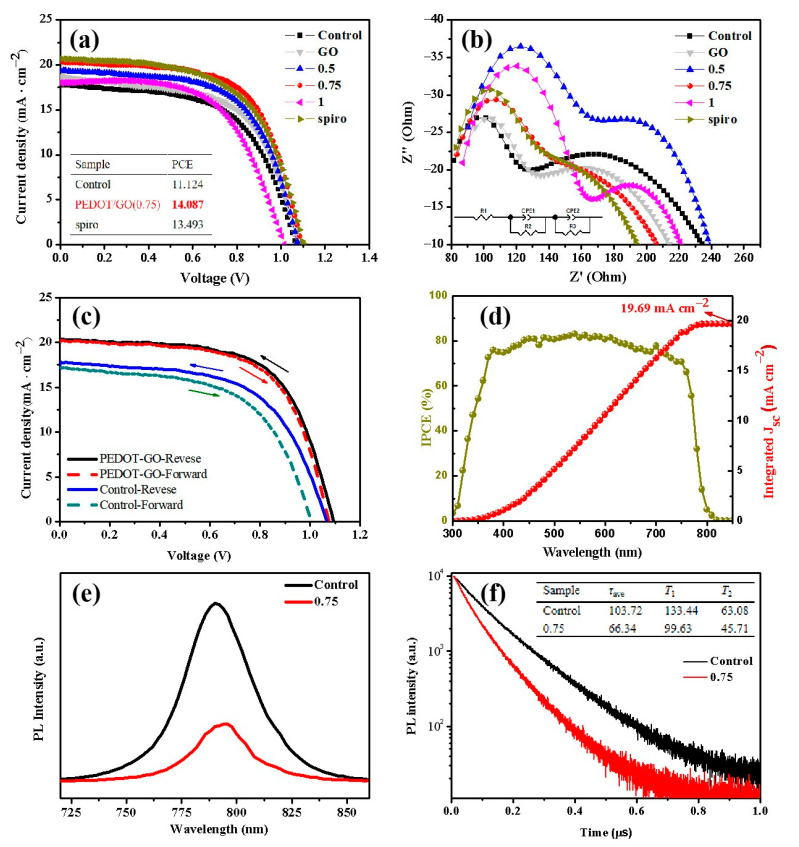
(**a**) J-V curves of control (HTL-free) sample and mass ratio PEDOT/GO: 0/1, 0.5/1, 0.75/1, 1/1, spiro-OMeTAD samples (**b**) Nyquist plots of resistance for the above samples (**c**) current-voltage characteristics with forward and reverse scans of PEDOT-GO and control sample (**d**) IPCE spectra of the PEDOT-GO (0.75) devices (**e**) steady-state PL spectra of PSCs of HTL-free sample and PSCs with PEDOT-GO (0.75) (**f**) time-resolved PL spectra of HTL-free sample and PSCs with PEDOT-GO (0.75).

In order to investigate the optimum component, samples with PEDOT/GO ratio as 0, 0.5, 0.75, 1 were prepared and tested, respectively. Figure 3a is the J–V curves of the mesoporous PSCs with different mass ratio PEDOT-GO composite films. The FTO/cp-TiO_2_/MAPbI_3_/C structure of PSC was fabricated as a control group compared with FTO/cp-TiO_2_/MAPbI_3_/PEDOT-GO (or spiro-OMeTAD)/C structure. Figure 3b shows the Nyquist plots and the equivalent circuit model of the PSCs [23]. The high frequency arc is reflected to the hole transport and extraction between the PEDOT-GO and the carbon cathode; the low frequency arc shows charge recombination of PSCs [24].

The corresponding photovoltaic parameters are shown in Table 1. The device exhibits the highest performance when the PEDOT-GO mass ratio is 0.75, the PCE reached the 14.09% with the voltage (**V_oc_**) as 1.10 V, the short-circuit current (**J_sc_**), as 20.36 mA/cm^2^, and the fill factor (FF) as 0.63. The PCE of the modified sample was increased by 26.6% compared to the HTL-free one. It is worth noting that, the FTO/TiO_2_/MAPbI_3_/spiro-OMeTAD/C sample with the PCE of 13.49%, Voc of 1.10 V, Jsc of 20.70 mA/cm^2^, and FF of 0.59 shows similar performance to the PEDOT-GO (0.75) sample. Meanwhile, the lowest Rtr value of the PEDOT-GO (0.75) sample as 23.9 Ω indicates the excellent charge transfer performance, and the highest Rrec value as 187.4 Ω indicates its best anti-recombination property among all cells [25]. When the PEDOT/GO mass ratio is lower than 0.75, the **J_sc_** increases with the PEDOT content, but the **V_oc_** remains unchanged, indicating that the hole transport performance of the composite material is effectively optimized and enhanced. However, when the PEDOT/GO mass ratio gets higher than 0.75, the dispersion of the composite material in the solvent becomes worse, suggesting the insufficient addition of GO, which leads to the deterioration of the film quality and negatively affects both the **J_sc_** and **V_oc_**. Finally, the mass ratio of PEDOT / GO is determined to be 0.75, the film quality and hole transport performance of the composites reach a balance, and the highest PCE is obtained. In addition, the hysteresis in the J-V curve of PEDOT-GO based devices is significantly reduced compared with the HTL-free devices (Figure 3c) [26]. PEDOT-GO composite material effectively optimized the hole extraction and transfer ability of the device, and reduces the built-in electric field at the interface of perovskite and HTL. Figure 3d shows the corresponding incident photon-to-electron conversion efficiency (IPCE) curves: the integrated current value for PEDOT-GO (0.75) sample was 19.69 mA cm^−2^, which is consistent with the **J_sc_** values extracted from the J-V curves.

The steady-state photoluminescence (PL) and the time-resolved photoluminescence (TRPL) tests were also conducted to evaluate the hole extraction ability of the HTL [27]. In Figure 3e, with the introduction of PEDOT-GO as HTL, the intense fluorescence at 790 nm is obviously suppressed. As shown in Figure 3f, for the control sample, the fast decay time (τ_1_) was 133.44 ns, and the slow decay time (τ_2_) was 63.08 ns, with an amplitude τ_ave_ (τ_ave_ = ΣA_i_τ_i_^2^/ΣA_i_τ_i_, where A_1_ and A_2_ are pre-exponential factors) of 103.72 ns. For the PEDOT-GO sample, τ_1_ was 99.63 ns, and τ_2_ was 45.71 ns, derived in an amplitude τave of 66.34 ns. Obviously, the sharp decrease in the average fluorescence lifetime indicates that the PEDOT-GO film effectively inhibits the charge recombination. This is consistent with the analysis of the polymer structure obtained by Raman. The above experiments further verify the positive effect of the PEDOT-GO film, which dramatically promote the separation and directional transmission of electrons and holes, thus explaining the increased PCE in the device [28].

**Figure 4:**
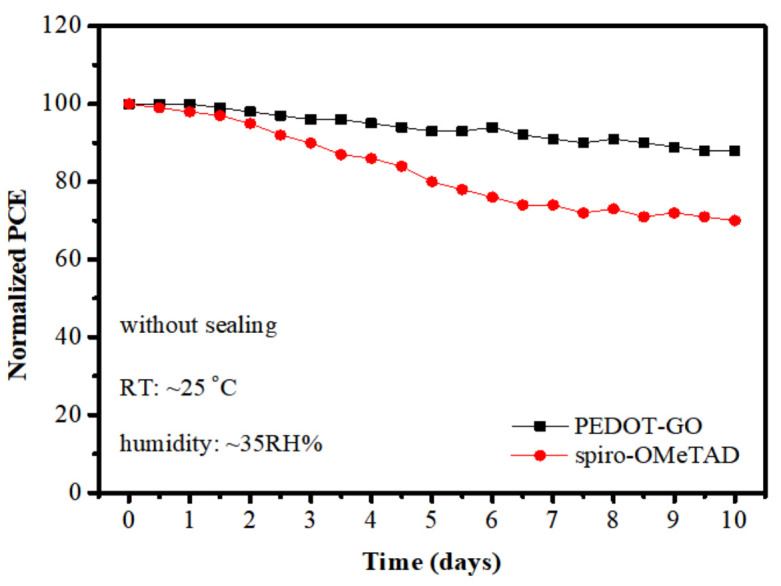
The durability test of spiro-OMeTAD-based solar cells and PEDOT-GO-based solar cells.

The stability of solar cells samples, without encapsulation, was further evaluated and compared in the air with the humidity of ~35% [29]. As shown in Figure 4, after ten-days placement, the PCE of spiro-OMeTAD-based solar cells decreased to 75% of the initial value, while the PCE of PEDOT-GO-based solar cells still maintained 90% of the initial value. As an approximation, the time at which the efficiency has degraded to 80% of the initial value was denoted as T_s80_ [30]. We can observe that the T_s80_ of spiro-OMeTAD-based solar cells was about 5 days, while it needed more than 10 days for that of PEDOT-GO-based solar cells. The device with PEDOT-GO HTL takes twice as long to fall to the same level of spiro-OMeTAD-based solar cells, which suggests that better durability can be realized by the introduction of PEDOT-GO composite films. 

## 4. Conclusions

In this paper, the PEDOT-GO composite film was successfully prepared as a hole transport layer for the PSCs. The functional thin film significantly inhibited the recombination of holes and electrons, improved the current density, and finally enhanced the PCE of the PSCs. With the mass ratio of 0.75, the highest PCE reaches 14.09%, which is 26% higher than that of the HTL-free sample, and similar results were obtained from Spiro-OMeTAD devices. Compared with Spiro-OMeTAD, PTAA, P3HT, and other traditional hole transport materials, it is much cheaper and more suitable for large-scale applications. 

## Figures and Tables

**Table 1:** Photovoltaic parameters of PSCs (Sweep speed of 0.25 V s^−1^, voltage range 0 V–1.2 V, electrode area of 0.06 cm^2^).

PEDOT/GO	Voc	Jsc	FF	PCE	Highest PCE	Rs	Rtr	Rrec
Control	1.05 ± 0.01	16.58 ± 1.15	0.61 ± 0.02	10.66 ± 0.46	11.12	72.05	74.12	95.13
0/1	1.05 ± 0.02	17.62 ± 1.11	0.61 ± 0.01	11.44 ± 0.57	12.01	51.06	23.23	175.1
0.5/1	1.06 ± 0.01	18.31 ± 1.09	0.62 ± 0.01	12.19 ± 0.6	12.79	53.4	30.02	140.7
0.75/1	1.09 ± 0.01	19.65 ± 0.72	0.63 ± 0.01	13.60 ± 49	14.09	52.3	23.9	187.4
1/1	0.99 ± 0.02	17.1 ± 0.95	0.57 ± 0.04	9.82 ± 1.12	10.94	75.04	76.82	80.54
Spiro	1.09 ± 0.01	20.20 ± 0.50	0.60 ± 0.01	13.18 ± 31	13.49	50.2	25.1	147.9

## Data Availability

Not applicable.

## References

[1] Song Z.N., McElvany C.L., Phillips A.B., Celik I., Krantz P.W., Watthage S.C., Liyanage G.K., Apul D., Heben M.J. (2017). A technoeconomic analysis of perovskite solar module manufacturing with low-cost materials and techniques. Energy Environ. Sci..

[2] Chen X., Xu G., Zeng G., Gu H.W., Chen H.Y., Xu H.T., Yao H.F., Li Y.W., Hou J.H., Li Y.F. (2020). Realizing Ultrahigh Mechanical Flexibility and >15% Efficiency of Flexible Organic Solar Cells via a Welding Flexible Transparent Electrode. Adv. Mater..

[3] Correa-Baena J.P., Tress W., Domanski K., Anaraki E.H., Turren-Cruz S.H., Roose B., Boix P.P., Grätzel M., Saliba M., Abate A. (2017). Identifying and suppressing interfacial recombination to achieve high open-circuit voltage in perovskite solar cells. Energy Environ. Sci..

[4] Jung E.H., Jeon N.J., Park E.Y., Moon C.S., Shin T.J., Yang T.Y., Noh J.H., Seo J.W. (2019). Efficient, stable and scalable perovskite solar cells using poly(3-hexylthiophene). Nature.

[5] Arora N., Dar M.I., Hinderhofer A., Pellet N., Schreiber F., Zakeeruddin S.M., Graetzel M. (2017). Perovskite solar cells with cuscn hole extraction layers yield stabilized efficiencies greater than 20%. Science.

[6] Zhao Q., Wu R., Zhang Z., Xiong J., He Z., Fan B., Dai Z., Yang B., Xue X., Cai P. (2019). Achieving efficient inverted planar perovskite solar cells with nondoped ptaa as a hole transport layer. Org. Electron..

[7] Han W., Ren G., Liu J., Li Z., Guo W., Bao H., Liu C., Guo W. (2020). Recent progress of inverted perovskite solar cells with a modified PEDOT:PSS hole transport layer. ACS Appl. Mater. Interfaces.

[8] Lian X., Chen J., Zhang Y., Tian S., Qin M., Li J., Andersen T.R., Wu G., Lu X., Chen H. (2019). Two-dimensional inverted planar perovskite solar cells with efficiency over 15% via solvent and interface engineering. J. Mater. Chem. A.

[9] Lin Y.J., Ni W.S., Lee J.Y. (2015). Effect of incorporation of ethylene glycol into PEDOT:PSS on electron phonon coupling and conductivity. J. Appl. Phys..

[10] Jiang X., Yu Z., Zhang Y., Lai J.B., Li J.J., Gurzadyan G.G., Yang X.C., Sun L.C. (2017). High-Performance Regular Perovskite Solar Cells Employing Low-Cost Poly(ethylenedioxythiophene) as a Hole-Transporting Material. Sci. Rep..

[11] Yu W., Wang K., Guo B., Qiu X., Hao Y., Chang J.J., Li Y. (2017). Effect of ultraviolet absorptivity and waterproofness of poly(3,4-ethylenedioxythiophene) with extremely weak acidity, high conductivity on enhanced stability of perovskite solar cells. J. Power Sources.

[12] Wu Z., Bai S., Xiang J., Yuan Z., Yang Y., Cui W., Gao X.Y., Liu Z., Jin Y.Z., Sun B.Q. (2014). Efficient planar heterojunction perovskite solar cells employing graphene oxide as hole conductor. Nanoscale.

[13] Wang M., Duan X., Xu Y., Duan X. (2016). Functional three-dimensional graphene/polymer composites. ACS Nano.

[14] Oger N., Lin Y.T.F., Labrugere C., le Grognec E., Rataboul F., Felpin F.X. (2016). Practical and scalable synthesis of sulfonated graphene. Carbon.

[15] Yu J.C., Hong J.A., Jung E.D., Kim D.B., Baek S.M., Lee S., Cho S., Park S.S., Choi K.J., Song M.H. (2018). Highly efficient and stable inverted perovskite solar cell employing PEDOT:GO composite layer as a hole transport layer. Sci. Rep..

[16] Zhao H., Zhao S.Q., Li Q., Khan M.R., Liu Y., Lu P., Huang C.X., Huang L.J., Jiang T. (2020). Fabrication and properties of waterborne thermoplastic polyurethane nanocomposite enhanced by the POSS with low dielectric constants. Polymer.

[17] Zhao H., She W., Shi D., Wu W., Zhang Q.C., Li R.K.Y. (2019). Polyurethane/POSS nanocomposites for superior hydrophobicity and high ductility. Compos. Part B Eng..

[18] Xue T.Y., Chen G.S., Hu X.T., Su M., Huang Z.Q., Meng X.C., Jin Z., Ma J., Zhang Y.Q., Song Y.L. (2021). Song.Mechanically Robust and Flexible Perovskite Solar Cells via a Printable and Gelatinous Interface. ACS Appl. Mater. Interfaces.

[19] Xu B., Gopalan S.A., Gopalan A.I., Muthuchamy N., Lee K.P., Lee J.S., Jiang Y., Lee S.W., Kim S.W., Kim J.S. (2021). Functional solid additive modified PEDOT:PSS as an anode buffer layer for enhanced photovoltaic performance and stability in polymer solar cells. Sci. Rep..

[20] Zhao H., Zhao S.Q., Hu G.H., Zhang Q.C., Liu Y., Huang C.X., Li W., Jiang T., Wang S.F. (2019). Synthesis and characterization of waterborne polyurethane/polyhedral oligomeric silsesquioxane composites with low dielectric constants. Polym. Adv. Technol..

[21] Wang Y., Hu Y., Han D., Yuan Q., Cao T., Chen N., Zhou D., Cong H., Feng L. (2019). Ammonia-treated graphene oxide and PEDOT:PSS as hole transport layer for high-performance perovskite solar cells with enhanced stability. Org. Electron..

[22] Zhou Y., Mei J., Feng J.J., Sun D.W., Mei F., Xu J.X., Cao X. (2020). Effects of PEDOT:PSS:GO composite hole transport layer on the luminescence of perovskite light-emitting diodes. RSC Adv..

[23] Cho J.S., Jang W., Mun S.C., Yi M.J., Park J.H., Wang D.H. (2018). Tuning surface chemistry and morphology of graphene oxide by γ-ray irradiation for improved performance of perovskite photovoltaics. Carbon.

[24] Liu X., Hu L., Wang R., Li J., Gu H., Liu S., Zhou Y., Tu G. (2019). Flexible perovskite solar cells via surface-confined silver nanoparticles on transparent polyimide substrates. Polymers.

[25] Wang Y., Wang S., Chen X., Li Z., Wang J., Li T., Deng X. (2018). Largely enhanced voc and stability in perovskite solar cells with modified energy match by couple 2D interlayers. J. Mater. Chem. A.

[26] Li R., Liu M.N., Matta S.K., Hiltunen A., Deng Z.F., Wang C., Dai Z.C., Russo S.P., Vivo P., Zhang H.C. (2021). Sulfonated Dopant-Free Hole-Transport Material Promotes Interfacial Charge Transfer Dynamics for Highly Stable Perovskite Solar Cells. Adv. Sustain. Syst..

[27] Kim M., Yi M., Jang W., Kim J.K., Wang D.H. (2020). Acidity Suppression of Hole Transport Layer via Solution Reaction of Neutral PEDOT:PSS for Stable Perovskite Photovoltaics. Polymers.

[28] Zhang H.C., Liu M.N., Yang W.J., Judin L., Hukka T.I., Priimagi A., Deng Z.F., Vivo P. (2019). Thionation Enhances the Performance of Polymeric Dopant-Free Hole-Transporting Materials for Perovskite Solar Cells. Adv. Mater. Interfaces.

[29] Ghadiri M., Kang A.K., Gorji N.E. (2020). XRD characterization of graphene-contacted perovskite solar cells: Moisture degradation and dark-resting recovery. Superlattices Microstruct..

[30] Canil L., Salunke J., Wang Q., Liu M.N., Köbler H., Flatken M., Gregori L., Meggiolaro D., Ricciarelli D., Angelis F.D. (2021). Halogen-Bonded Hole-Transport Material Suppresses Charge Recombination and Enhances Stability of Perovskite Solar Cells. Adv. Energy Mater..

